# Multiple Antibody Targets on Herpes B Glycoproteins B and D Identified by Screening Sera of Infected Rhesus Macaques with Peptide Microarrays

**DOI:** 10.1371/journal.pone.0086857

**Published:** 2014-01-31

**Authors:** Sven-Kevin Hotop, Ahmed Abd El Wahed, Ulrike Beutling, Dieter Jentsch, Dirk Motzkus, Ronald Frank, Gerhard Hunsmann, Christiane Stahl-Hennig, Hans-Joachim Fritz

**Affiliations:** 1 Unit of Infection Models, German Primate Center, Göttingen, Germany; 2 Department of Virology, University Medical Center, Georg-August University Göttingen, Göttingen, Germany; 3 Department of Chemical Biology, Helmholtz Centre for Infection Research, Braunschweig, Germany; 4 Courant Research Centre Geobiology, Göttingen, Germany; University of Pennsylvania School of Veterinary Medicine, United States of America

## Abstract

Herpes B virus (or *Herpesvirus simiae* or *Macacine herpesvirus 1*) is endemic in many populations of macaques, both in the wild and in captivity. The virus elicits only mild clinical symptoms (if any) in monkeys, but can be transmitted by various routes, most commonly *via* bites, to humans where it causes viral encephalitis with a high mortality rate. Hence, herpes B constitutes a considerable occupational hazard for animal caretakers, veterinarians and laboratory personnel. Efforts are therefore being made to reduce the risk of zoonotic infection and to improve prognosis after accidental exposure. Among the measures envisaged are serological surveillance of monkey colonies and specific diagnosis of herpes B zoonosis against a background of antibodies recognizing the closely related human herpes simplex virus (HSV). 422 pentadecapeptides covering, in an overlapping fashion, the entire amino acid sequences of herpes B proteins gB and gD were synthesized and immobilized on glass slides. Antibodies present in monkey sera that bind to subsets of the peptide collection were detected by microserological techniques. With 42 different rhesus macaque sera, 114 individual responses to 18 different antibody target regions (ATRs) were recorded, 17 of which had not been described earlier. This finding may pave the way for a peptide-based, herpes B specific serological diagnostic test.

## Introduction

Herpes B was first described as a human case of acute ascending myelitis with lethal outcome, following a monkey bite; a filterable transmitting agent (“B virus”, later named herpes B virus) could be recovered *post mortem* from various tissues of the victim [1]. Herpes B belongs to the genus *Simplexvirus* within the *Alphaherpesvirinae* subfamily of the *Herpesviridae* family. The enveloped particle harbors a linear double-stranded DNA genome of approximately 157 kb [2]–[4]. The genomes of herpes B and human herpes simplex virus type 1 (HSV-1) and type 2 (HSV-2) are made up to a great extent by homologous genes in the same order and orientation [4]. This similarity at the level of genome organization is reflected by an average of approximately 62% identity of amino acid residues across all homologous proteins herpes B shares with both HSV-1 and HSV-2 [2]. The viral envelope contains twelve glycoproteins, among them four with proven high immunogenicity: gB, gC, gD and mgG [4], [5].

Establishment and sustainable maintenance of specific pathogen-free monkey colonies depends on reliable identification of carrier animals [6]. With herpes B, this cannot be achieved by testing body fluids for virus-specific nucleic acids, due to long periods of latency during which the viral genome only persists in sensory ganglia but no virions are shed to the periphery [4]. Thus, serological identification of anti-herpes B antibodies is the only way to discover infected animals. Several serological techniques relying on recombinant proteins and cell lysates of herpes B are currently in use to detect this viral infection [5]. In addition, a commercially available herpes simplex virus (HSV) ELISA has been employed because of cross-reactivity [7]. By using this assay, however, differences in primary sequences could lead to “false negative” results which must be prevented for safety reasons. For this purpose, mapping anti-herpes B antibody targets may be a valuable tool to improve herpes B diagnosis. To date, only one epitope has been described at the C-terminal end of gD [8]. Whether or not other epitopes exist has been unknown until now.

To elucidate the spatial distribution of BV epitopes we have developed and applied a peptide microarray covering the whole amino acid sequences of the structural proteins gB and gD. Using this high throughput platform we have identified seventeen novel linear epitopes in addition to the one that was previously described [8]. The study provides evidence that profiling of sera at single epitope resolution is a powerful tool to comprehensively describe individual B cell responses that could significantly improve current diagnostics.

## Materials and Methods

For the collection of samples for diagnostic purposes, we did not need an IACUC approval because this was not related to any experimental procedure but was linked to animal care. However, the German Primate Center has the permission to breed and keep nonhuman primates (as stated in the paragraph below) which includes animal health monitoring and sampling for diagnostic purposes.

### Origin of Monkey Sera, Ethics Statement and Animal Care

Thirty-six test sera used in this study were derived from blood samples collected by experienced veterinarians from rhesus macaques (*Macaca mulatta*) as part of the breeding colony at the German Primate Center (DPZ). Sampling was conducted within the framework of the annual animal health monitoring for a variety of veterinary diagnostics. For the collection of the blood samples animals were anesthetized intra-muscularly with 10–15 mg ketamine per kg body weight; none of the animals were euthanized. The samples were cryopreserved as part of the routine sample banking of the Unit of Animal Husbandry at the DPZ. According to §11 of the German Animal Welfare Act, the DPZ has permission to breed and house nonhuman primates under license number 392001/7 issued by the local veterinary authorities. Animals were cared for by trained staff at the DPZ in accordance with institutional guidelines based on the German Animal Welfare Act. This comprises supervision and advice by the institutional animal welfare officer. The animals were housed under conditions as stipulated in the European Directive 2010/63/EU (directive on the protection of animals used for experimental and other scientific purposes) and the EU Recommendations 2007/526/EG (guidelines for the accommodation and care of animals used for experimental and other scientific purposes) which are in line with the NRC (USA) Guide for the Care and Use of Laboratory Animals. The macaques had access to indoor (43 m^2^ and a height of 7 m, room temperature 18–20°C) and outdoor areas (a height of 7 m and floor space of 370 m^2^ with a natural surface), equipped with wooden climbing gadgets, swings, ropes and chains, fire hoses, car tires, barrels and water basins. Meals were supplied three times a day in different compositions where the basic food supply consists of dry monkey biscuits and protein-enriched mash supplemented with fresh fruits and vegetables and additional edible items (e.g. seeds, cereals, nuts). Fresh water was provided *ad libitum*.

### Reference Sera

Three anti-herpes B positive reference sera and three negative ones were kindly provided by the National B Virus Resource Laboratory, Atlanta, GA, USA. The positive monkey sera were derived from naturally infected rhesus macaques. The reference sera had been identified as positive/negative by an ELISA based on whole lysed herpes B antigen reaching titers of around 1∶10,000. The positive responses were confirmed by Western Blot and second ELISA in which gB, gC, gD and mgG, produced by genetic engineering, were used for coating (M.J. Wildes, personal communication).

### Peptide Microarray

**Figure 1:**
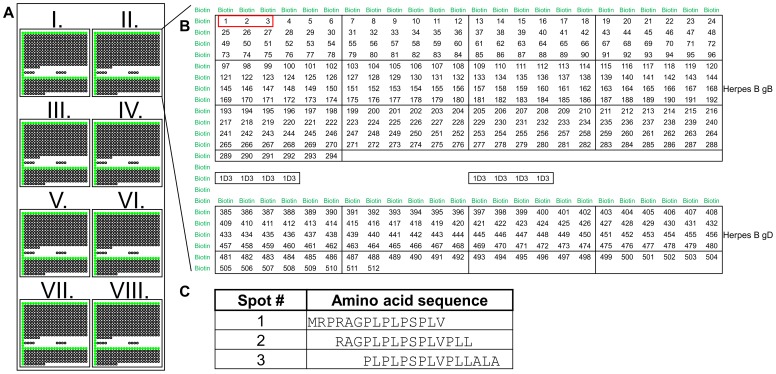
Peptide microarray layout. (A) Each chip contains eight arrays (I–VIII) with identical peptide sequence and layout. Peptides were printed at three different amounts per spot, 360 fmol (I and II), 180 fmol (III–VI), and 90 fmol (VII and VIII). For orientation, each array is bordered by marker spots (biotin) that give rise to green fluorescent signals upon screening. (B) The upper part of each array consists of 294 15mers corresponding to the amino acid sequence of gB, each overlapping by 12 amino acid residues. The lower part is printed with peptides corresponding to the sequence of gD with the same specifications as for gB. The empty space between the gB and gD peptides was used for spotting of process control peptides (1D3). (C) Amino acid sequence of the first three peptides covering gB, corresponding to spots #1–#3 in (B).

Herpes B scanning chips, based on gB and gD of herpes B, strain E2490 (GenBank accession number AF533768), were used for screening the monkey sera. Peptides were synthesized as described [9], [10]. A detailed description of the microarray layout is given in Figure 1.

### Data Acquisition

The chip was washed with 98% [v/v] ethanol for 3 min and then three times with Tris-buffered saline (TBS: 240 mM Tris/HCl, 1.5 mM KCl, 470 mM NaCl, pH 7.0) for 3 min each. After incubation with blocking buffer (2% [w/v] casein in T-TBS: TBS, 0.05% [w/v] Tween 20) at room temperature for one hour, the chip was washed once with T-TBS for 3 min. Thereafter, 60 µl of serum sample (1∶60 in blocking buffer) was loaded onto the chip. The chip was incubated in a humidified chamber at 4°C overnight. Thereafter, the chip was washed three times for 5 min each with T-TBS. To visualize bound antibodies and biotin spots, 60 µl of blocking buffer containing monkey IgG cross-reactive Cy5-conjugated goat anti-human IgG and Cy3-conjugated mouse anti-biotin antibody (Jackson ImmunoResearch, USA) were added to the chip at optimal dilutions. The chip was kept in the above-mentioned chamber at room temperature for 1.5 hours. Subsequently, the chip was washed twice for 5 min each with T-TBS, three times with distilled water for 5 min each, and dried in a stream of compressed air. The distribution of fluorescence intensities across the chip was determined using a DNA microarray scanner (Agilent Technologies, USA). Enrichment of IgG antibodies was done with a protein A IgG purification kit according to the manufacturer’s instructions (Pierce Biotechnology, Rockford, USA).

### Algorithm for Semi-automated Spot Calling

Experimentally obtained fluorescence patterns were displayed using Agilent Feature Extraction program, Version 7.5 (Agilent Technologies, USA) in standard settings and saved as a bitmap containing 8 bit of brightness information per pixel for each of the three color channels. Bitmaps (without separation of colors) were manually aligned (tilt angle, x/y-translation) with a pre-defined grid of adjoining squares using CorelDRAW X5 software (Corel Inc., USA) such that, in accord with the spot layout of the chip and the resolution of the chip reader, each square had a size of 20×20 pixels and housed a single spot. Subsequently, readings of red and green fluorescence (test and marker spots, respectively) were separated using ImageJ software [11]. For numerical processing of test spot readings, all corresponding sets of 400 numbers were summed up individually and the resulting values were exported to Excel (Microsoft, version 2010). For each individual array (eight per chip, see Figure 1), the mean of all cell readings was computed and spots with values above that average were discarded as potential positive values. From the remaining subset, a second average was calculated and a final cut-off was defined as the second average plus one standard deviation. Spots in the “above cut-off” class were defined as a positive response if they were above the threshold in at least six out of eight arrays.

### HSV-ELISA

An *anti*-herpes simplex virus ELISA (Enzygnost Anti-HSV/IgG Test Kit, DADE Chiron, Germany), known to be cross-reactive with herpes B antibodies [7], was used according to the manufacturer’s instructions with the following modification: a conjugate of goat *anti*-human IgG (H+L) and horseradish peroxidase (Jackson ImmunoResearch, West Grove, PA, USA) was employed because, in our hands, its sensitivity and performance proved superior to both the immunoconjugate included in the original kit and the one based on an *anti*-(simian) IgG antibody described earlier [7].

## Results

### Peptide Microarray Assay Design

**Figure 2:**
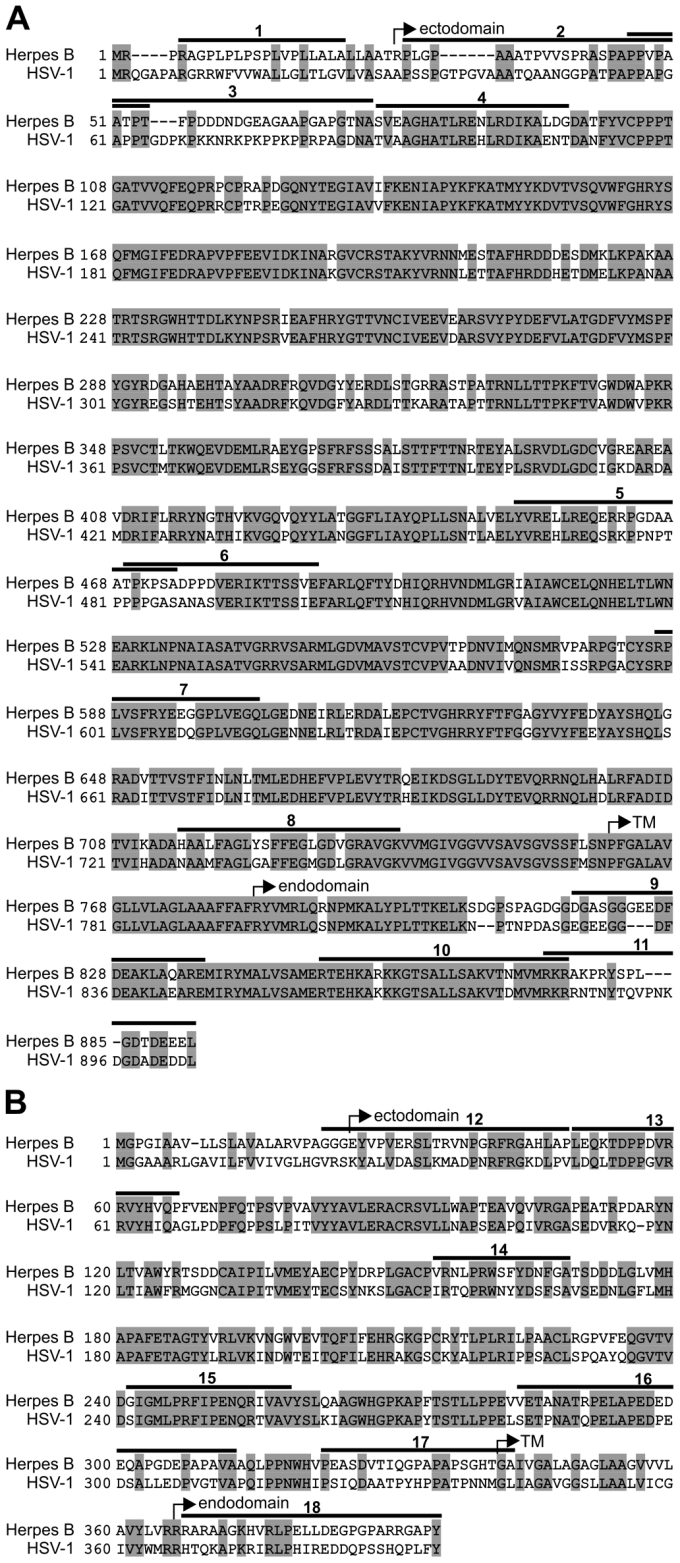
Aligned amino acid sequences of gB and gD from herpes B virus and HSV. Panel A: Glycoprotein B from herpes B (NP_851887) and herpes simplex virus type 1 (NP_044629); Panel B: Glycoprotein D from herpes B (NP_851925) and herpes simplex virus type 1 (NP_044668). Residue identity is highlighted in grey. Bars and numbers indicate antibody target regions (ATRs) identified in this study. The start of the ectodomain, the transmembrane region (TM), and the endodomain are indicated.

With the aim of serologically profiling macaque sera for footprints of herpes B antigens at single epitope resolution, peptide microarrays based on the highly immunogenic proteins gB and gD [5], [12] were prepared as described [9], [10]. The respective amino acid sequences and their alignments to the corresponding HSV-1 homologs are displayed in Figure 2. Protein gB shares 79.0% sequence identity with its HSV-1 homolog and 80.4% with that of HSV-2. For gD the corresponding values are 57.0% and 59.0%, respectively [2].

Sequences of 422 pentadecapeptides were designed to cover gB and gD of herpes B in an overlapping fashion (Figure 1). The peptides were prepared by ‘Spot-Synthesis’ [9] and printed onto glass slides [10]. The resulting microarray chips were used to analyze antibody spectra of rhesus macaque sera (6 references and 36 test samples).

In brief, chips were incubated with diluted macaque serum, followed by incubation with a cross-reactive secondary antibody (anti-human IgG) conjugated to a fluorescent dye. A standard chip reader was used for signal detection. Unprocessed readings (i.e. data as provided by the instrument as TIF files) were inspected visually, then analyzed numerically by a computed ‘spot calling’ algorithm described under Materials and Methods. All assignments of a positive or negative response to an individual array spot followed the output of this algorithm.

### General Features of Primary Data

**Figure 3:**
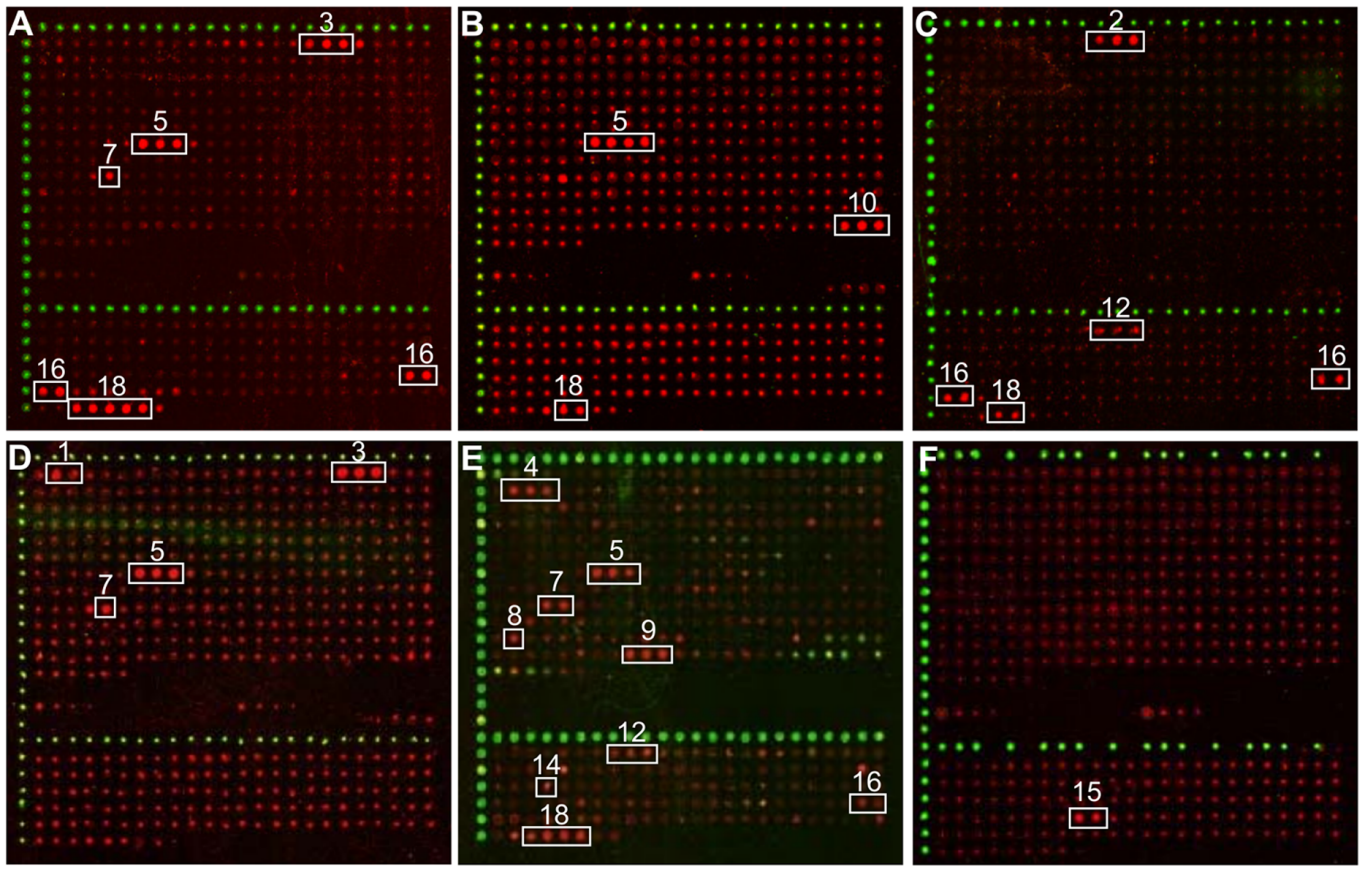
Graphical representation of results obtained with six different sera (single array enlargements). A is sample #11; B, #21; C, #16; D, #15; E; #4; F, #42. White frames indicate array spots identified as positive by the spot calling algorithm.

Application of a large set of sera from rhesus macaques resulted in a variety of positive signals on the microarray slides. Representative raw data obtained with sera from different animals are outlined in Figure 3. Spots identified as positive by the spot calling algorithm are indicated by a white frame. Some immediately conspicuous features of the data include the following. *(i)* In all experiments, positive spots make up a small subset. *(ii)* Positive spots are typically clustered as local, consecutive series. *(iii)* One multiply occurring response (ATR7) was represented in the majority of cases by an isolated single spot (numbered ‘7’ in Figure 3 A, D; compare to E). This exception is dealt with in detail below (see “Mapping ATRs to the three-dimensional structures of gB”). *(iv)* Overall, sera that were tested positive in the HSV-ELISA (Table 1) typically responded with several spot series on the peptide microarray. *(v)* Response spectra of individual sera, while having some overlap, differed from one another.

**Table 1:** Distribution of individual ATRs in sera of 42 different macaques.

Sample No.*	Antibody target region																		HSV-ELISA
	1	2	3	4	5	6	7	8	9	10	11	12	13	14	15	16	17	18	
	glycoprotein B											glycoprotein D							
1^1^																		**X**	pos.
2			**X**		**X**						**X**							**X**	pos.
3					**X**		**X**											**X**	pos.
4				**X**	**X**		**X**	**X**	**X**			**X**		**X**		**X**		**X**	pos.
5		**X**	**X**				**X**			**X**	**X**							**X**	pos.
6			**X**						**X**		**X**					**X**	**X**	**X**	pos.
7			**X**		**X**		**X**		**X**		**X**							**X**	pos.
8			**X**		**X**				**X**					**X**		**X**		**X**	pos.
9		**X**	**X**				**X**		**X**							**X**		**X**	pos.
10 ^2^		**X**	**X**		**X**		**X**					**X**						**X**	pos.
11			**X**		**X**		**X**									**X**		**X**	pos.
12			**X**		**X**				**X**							**X**		**X**	pos.
13			**X**				**X**									**X**		**X**	pos.
14		**X**	**X**		**X**											**X**			pos.
15	**X**		**X**		**X**		**X**												pos.
16		**X**										**X**				**X**		**X**	pos.
17					**X**		**X**									**X**		**X**	pos.
18		**X**										**X**						**X**	pos.
19					**X**											**X**		**X**	pos.
20						**X**						**X**	**X**						pos.
21					**X**					**X**								**X**	pos.
22																**X**		**X**	pos.
23		**X**					**X**												pos.
24		**X**																**X**	pos.
25																**X**		**X**	pos.
26																**X**		**X**	pos.
27												**X**						**X**	pos.
28								**X**											pos.
29																		**X**	pos.
30					**X**														pos.
31 ^1^																		**X**	pos.
32 ^1^																		**X**	pos.
33																			pos.
34																			neg.
35																			neg.
36																			neg.
37																			neg.
38																			neg.
39																			neg.
40																			neg.
41																			neg.
42															**X**				neg.

**Figure 4:**
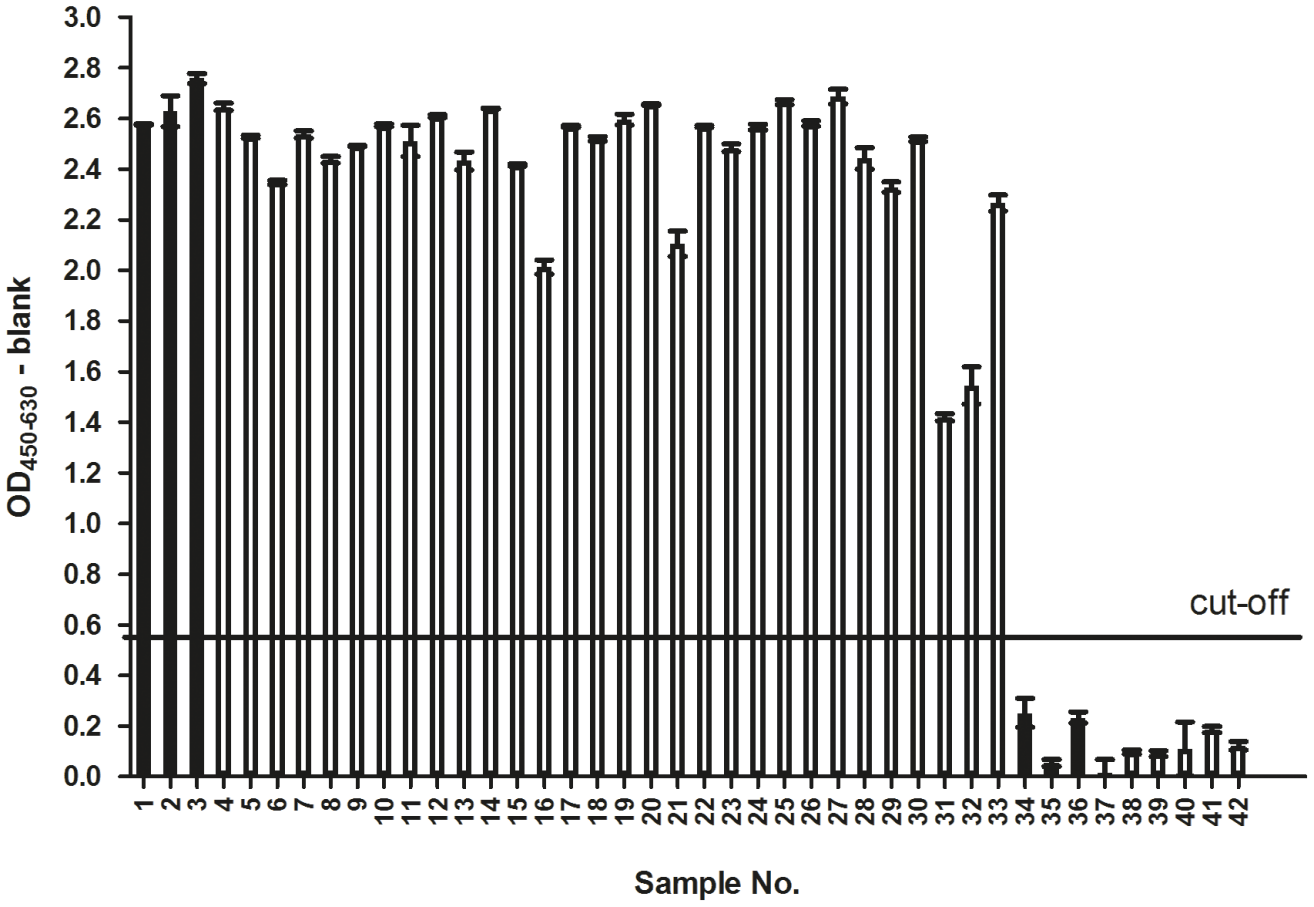
Graphical representation of results obtained with modified commercial *anti*-HSV ELISA. In total 42 serum samples were analyzed. The black bars indicate positive (samples 1–3) and negative (samples 34–36) references; Source: National B Virus Resource Laboratory, Atlanta, USA. Open bars represent samples derived from the DPZ rhesus macaque breeding groups. The cut-off value was defined as twice the value of the reaction of the negative reference sample (# 34) yielding the highest O.D. among the three negative reference samples and is indicated by a horizontal line. Sera were tested at a 1∶200 dilution. Standard deviations (SD) of duplicate measurements are indicated.

* Serum samples were obtained in 2010 from rhesus macaques kept at the German Primate Center. Responses to particular ATRs are indicated (X). Column designated “HSV-ELISA”: Results of HSV-ELISA assay (Figure 4).

^1^ Results after affinity enrichment of IgG using protein A (samples of crude sera produced high background).

^2^Results from serum of the same animal retrieved in 2008 (sample of 2010 produced high background). Numbers of positive (1–3) and negative sera (34–36) are given in bold; source: National B Virus Resource Laboratory, Atlanta, GA, USA.

According to the experimental design, a consecutive series of positive spots represents the binding area of a single or multiple antibodies that are present in the serum. The epitope length becomes smaller with increasing number of positive spots and, under the chosen regime of tiling pentadecapeptides with a progression of three amino acid residues per step, the maximal number of spots in one series is five. Since distinct antibodies can contribute to one spot series we use the term ‘antibody target region’ (ATR).

### Synopsis of Total Data Set

A total of 42 sera were investigated. Columns marked “1” to “18” in Table 1 list contributions of different ATRs to a total of 114 individual positive reactions. With 63 responses elicited by gB and 51 by gD, the two proteins are of comparable antigenicity, at least with regard to the detected linear epitopes. The study is grounded in six reference sera obtained from an external source (see Materials and Methods) and pre-marked by the supplier as positive (samples 1–3) or negative (samples 34–36). These +/− assignments were reflected by own HSV-ELISA experiments (rightmost column in Table 1 and Figure 4).

The 36 test sera derived from different rhesus macaque breeding groups which had been known to include carriers of herpes B. By the HSV-ELISA criterion, 30 test sera were classified as positive, six as negative. In the parallel chip assay, one of the HSV-ELISA-positive sera (sample 33) did not respond to any of the 18 gB and gD ATRs; likewise, in the HSV-ELISA-negative group, one serum (sample 42) elicited a positive response on the chip (ATR15). In all other cases the results of one assay matched that of the other.

### Response Statistics

**Figure 5:**
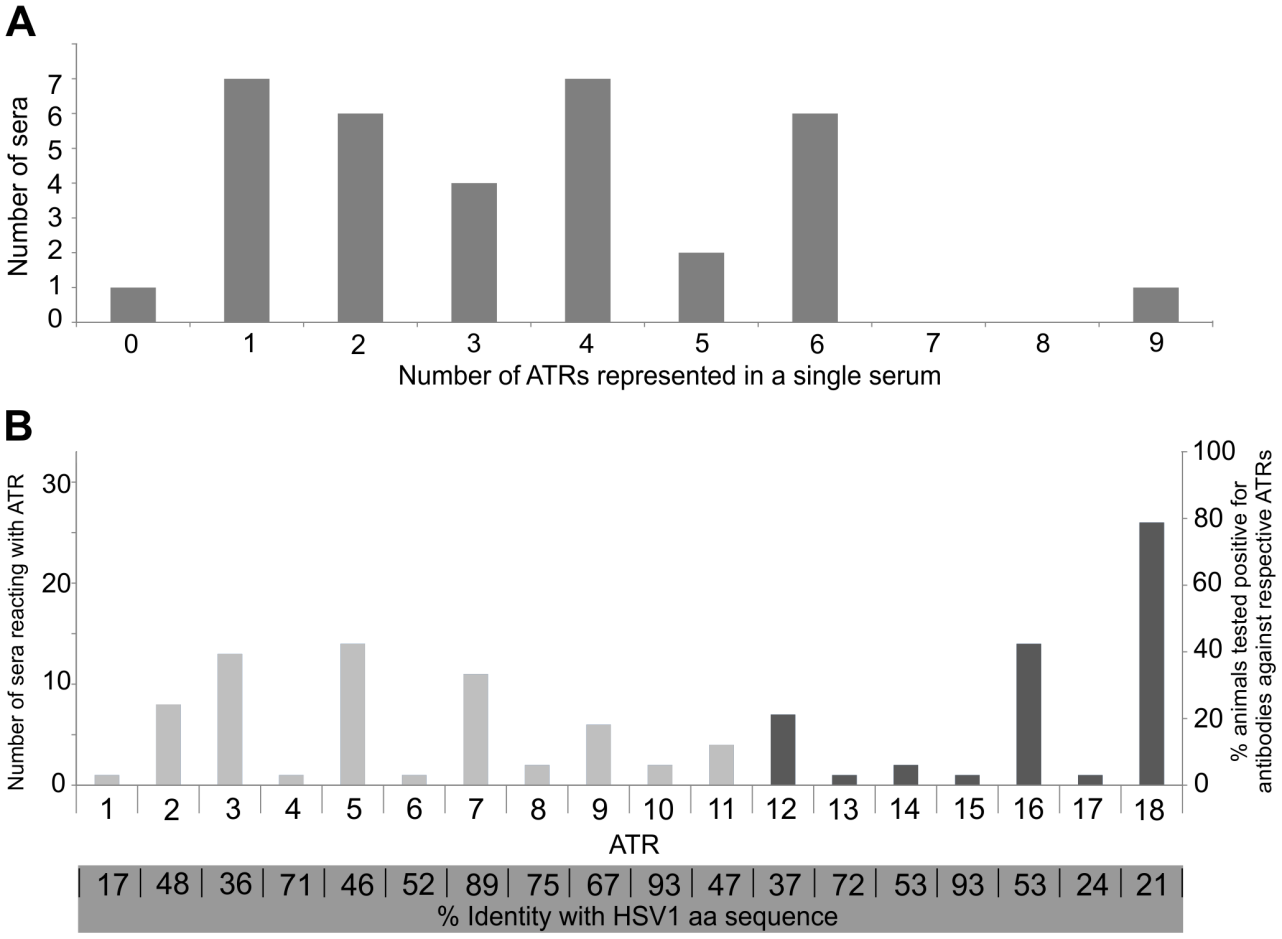
Statistics of ATR occurrence. 114 individual responses observed in 34 sera (sample numbers 1 to 33 and 42, also compare Table 1). PANEL A: Distribution of numbers of ATR responses seen in individual animals. PANEL B: Number of observed occurrences of individual ATRs (light grey: gB, dark grey: gD) and their respective percent identity with aligned sequence stretches of HSV-1 (bottom).

The 114 positive ATR responses are distributed unequally among sera derived from 34 macaques (HSV-ELISA-positive subset plus sample 42) corresponding to an average of 3.4 ATRs per serum. The distribution around this mean value is illustrated in Figure 5, panel A. The number of occurrences of individual ATRs is conspicuously different, varying between one (ATR1, 4, 6, 13, 15, 17 and 42) and 26 (ATR18). The complete spectrum of occurrences is shown in Figure 5, panel B. Of note, ATR18 was detected in 79% of all tested sera reacting with gD which is in the same range as reported [8]. Interestingly, we also found that in five ATR18-positive sera, the response was limited to peptides representing amino acid sequence immediately following the TM-domain (Table 2) and not extending into the previously described epitope [8].

**Table 2:** Break down of individual responses to ATR18 in monkey sera.

Sample No.	Spot No.					
	507	508	509	510	511	512
1		**X**	**X**			
2			**X**	**X**	**X**	
3			**X**	**X**		
4	**X**	**X**	**X**	**X**		
5		**X**	**X**	**X**	**X**	
6			**X**	**X**		
7		**X**	**X**	**X**	**X**	**X**
8	**X**	**X**	**X**			
9		**X**	**X**	**X**	**X**	**X**
10		**X**	**X**			**X**
11	**X**	**X**	**X**	**X**	**X**	
12		**X**		**X**		
13		**X**	**X**	**X**		
16		**X**	**X**			
17			**X**			
18		**X**	**X**	**X**	**X**	**X**
19	**X**	**X**	**X**	**X**		**X**
21			**X**	**X**		
22	**X**	**X**	**X**	**X**		
24			**X**	**X**		
25		**X**	**X**	**X**	**X**	
26			**X**	**X**	**X**	**X**
27		**X**				
29		**X**	**X**	**X**	**X**	
31					**X**	**X**
32		**X**				

Antibody binding with single peptides of ATR18 on the peptide microarray are indicated (X).

In the HSV-ELISA-positive subset (33 sera), there are nine samples without a gB response (27%). The respective number for gD is five (15%). Under the assumption of such response failures occurring independently, the expected rate of double failures amounts to 4%. There is, indeed, one case (sample 33) in which an HSV-ELISA-positive sample failed to react with any peptide derived from either gB or gD, which corresponds to a double failure rate of 3%. This discrepancy between HSV-ELISA and herpes B-specific peptide microarray could be attributable to antibodies directed against discontinuous or glycosylated epitopes or glycoproteins other than gB or gD. In one case we detected one positive response in the peptide microarray (ATR15/serum sample #42), while the same serum was tested negative by HSV-ELISA. Whether this represents a ‘false negative’ in HSV ELISA or ‘false-positive’ result in the chip assay cannot be judged, unless proven by a third independent method.

### Mapping ATRs to the Three-dimensional Structure of gB

**Figure 6:**
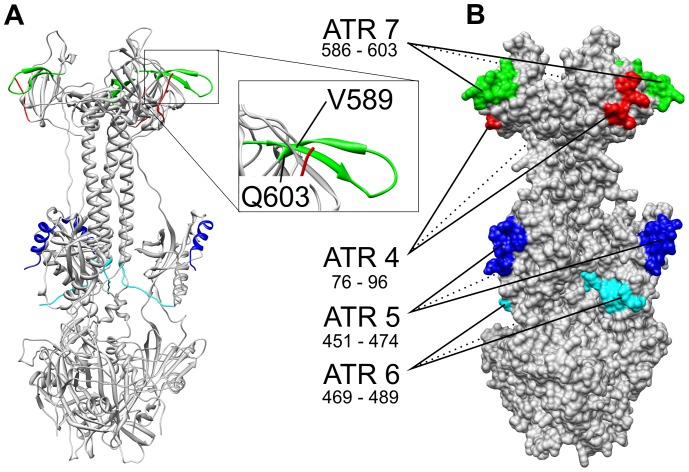
Alignment-guided mapping of four ATRs to the three dimensional structure of an HSV-1 gB ectodomain fragment trimer (PDB number 3NWA chain A residues D103 to A730). Compared to the unprocessed translation product described in Figure 2A, the crystallized fragment lacks the 102 N-terminal residues plus the entire C-terminal part (residues 731–904 spanning the carboxyterminal end of the ectodomain: residues 731–773, the transmembrane region: residues 774–795 and the endodomain: residues 796–904). Within the crystallized fragment, there are two stretches of disordered chains (residues 477–491 and 724–730 [14]). Panel A shows the molecule in ribbon representation and panel B as solvent-accessible surface. ATR4 sequence (7 most carboxyterminal residues) is shown in red, ATR5 (14 N-terminal residues) in blue, ATR6 sequence (eleven most carboxyterminal residues) is highlighted in cyan and ATR7 in green. The ribbon representation of ATR7 is magnified in the inset to emphasize the β-hairpin corresponding to spots #196 and197 of the peptide microarray.

*Simplexvirus* glycoprotein B is an integral membrane protein, anchored in the viral envelope with one membrane-spanning domain [13]. While no direct structural information is available for herpes B gB, the three-dimensional structure of a soluble ectodomain fragment of its closely related HSV-1 homolog has been determined by x-ray crystallography [14]. According to the sequence alignment (Figure 2), four out of 11 ATRs (# 4, 5, 6 and 7) of herpes B gB identified in this present study map within the crystallized HSV-1 fragment. Projection of the four ATRs onto the crystal structure revealed that they were located on the protein surface (Figure 6).

A response to ATR6 was observed only once in the entire study (sample 20, Table 1) whereas antibodies directed against ATR7 were detected in ten sera (Figure 5, panel B; Table 1). There are two classes of ATR7 response: In one case (sample 4, Figure 3E) two peptides (array spots #196 and #197) were detected, together defining an ATR length of 18 residues as indicated in Figure 2 and Table 3. In the other nine cases, the ATR7 response was restricted to a single responding peptide (# 197; Figure 3A and D), a result formally implying an epitope comprising 15 amino acid residues (V589 to Q603, Figure 2 and Figure 6). A structural interpretation of this unexpected finding is given under Discussion.

**Table 3:** Sequence, length, number of spots, and position within herpes B sequence of the 18 ATRs.

ATR	Spot no.	aa position	Sequence	aa length
**glycoprotein B**				
1	2–3	4–21	RAGPLPLPSPLVPLLALA	18
2	10–14	28–54	PLGPAAATPVVSPRASPAPPVPAATPT	27
3	16–21	46–75	PPVPAATPTFPDDDNDGEAGAAPGAPGTNA	30
4	26–28	76–96	SVEAGHATLRENLRDIKALDG	21
5	151–154	451–474	YVRELLREQERRPGDAAATPKPSA	24
6	157–159	469–489	TPKPSADPPDVERIKTTSSVE	21
7	196–197	586–603	RPLVSFRYEEGGPLVEGQ	18
8	239–242	715–738	HAALFAGLYSFFEGLGDVGRAVGK	24
9	273–275	817–837	DGASGGGEEDFDEAKLAQARE	21
10	284–288	850–876	RTEHKARKKGTSALLSAKVTNMVMRKR	27
11	292–294	874–892	RKRAKPRYSPLGDTDEEEL	19
**glycoprotein D**				
12	392–396	22–48	GGGEYVPVERSLTRVNPGRFRGAHLAP	27
13	401–402	49–66	LEQKTDPPDVRRVYHVQP	18
14	436	154–168	VRNLPRWSFYDNFGA	15
15	465–466	244–258	GIGMLPRFIPENQRIVAV	18
16	479–484	283–312	VETANATRPELAPEDEDEQAPGDEPAPAVA	30
17	492–494	322–342	PEASDVTIQGPAPAPSGHTGA	21
18	507–512	367–394	RARAAGKHVRLPELLDEGPGPARRGAPY	28

### Mapping ATRs to the Three-dimensional Structure of gD

**Figure 7:**
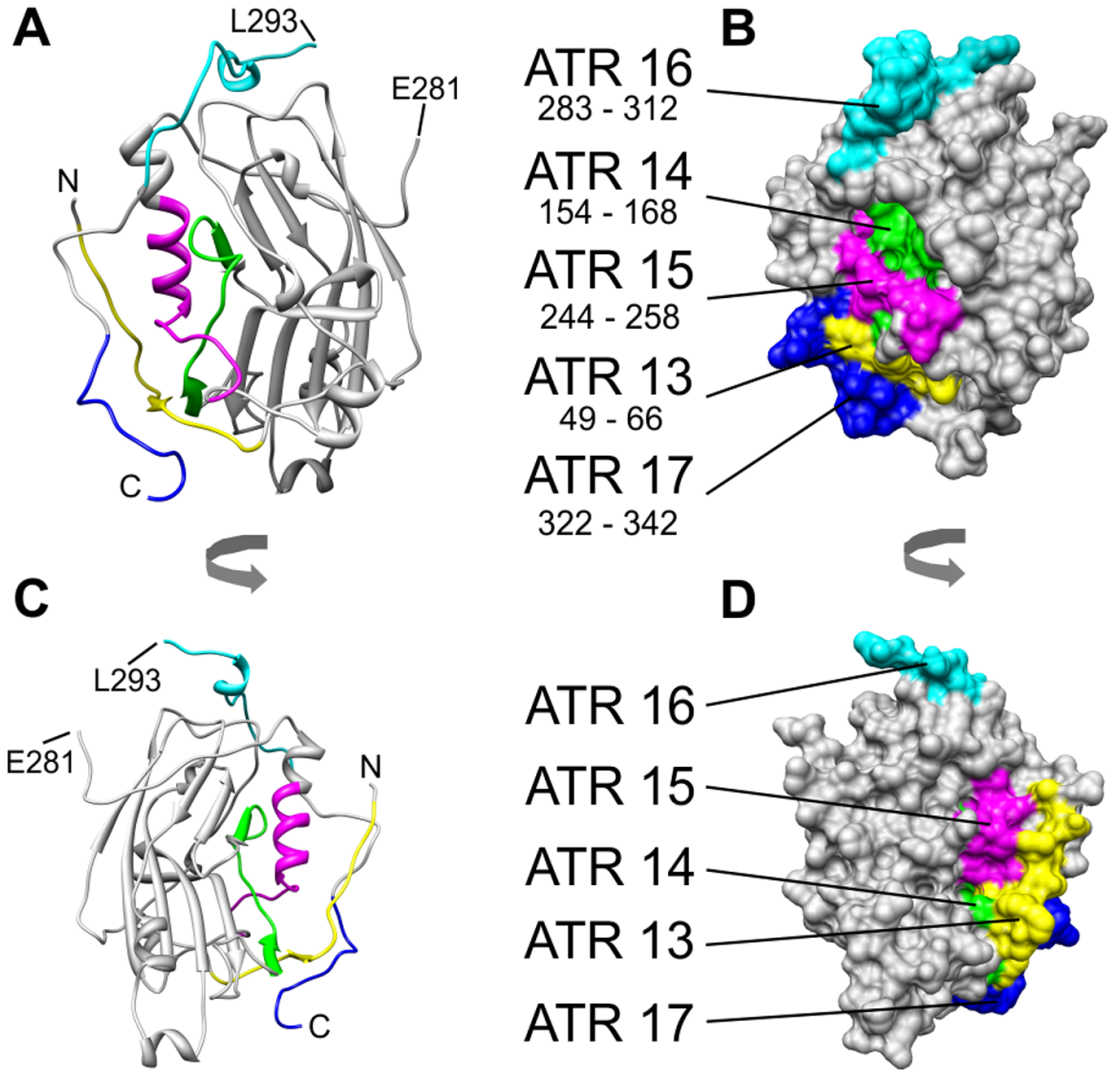
Alignment-guided mapping of ATR13–17 to the three-dimensional structure of the HSV-1 gD ectodomain fragment gD306_307C_ (PDB number 2C36, chain A). Applying the numbering scheme of Figure 2B, the molecule would be called gD332C. Compared to the unprocessed translation product illustrated in Figure 2B, the crystallized fragment lacks the 25 N-terminal residues (secretion leader) plus the entire C-terminal part (residues 342–394) which comprises the transmembrane region (residues 342–364) and the endodomain (residues 365–394). In addition, the following polypeptide stretches are missing from the PDB file: Residues 26–46, residues 282–292 and residues 333–341 (for commentary refer to [15]). In panels A and C, start and end of polypeptide chain with well-defined structure are indicated by letters N and C and the residues bordering the internal disordered segment as E281 and L293. Pairs of panels (A/B and C/D) show the molecule in the same orientation in ribbon representation and as solvent-accessible surface. The two pairs are related to one another by a 180° rotation about the vertical molecule axis. Color code is as follows: yellow: ATR13, green: ATR14, magenta: ATR15, cyan: ATR16 (20 most carboxyterminal residues), blue: ATR17 (11 most aminoterminal residues).

Like gB (compare above), *Simplexvirus* gD is anchored in the viral envelope by a single membrane-spanning domain [15]. The three-dimensional structure of a soluble fragment of HSV-1 gD (“gD306_307C_”, PDB number 2C36, chain A) has been determined by x-ray crystallography [15]. Of the seven ATRs of herpes B gD detected by serological chip assay, ATR18 is not contained in the crystallized fragment; ATR12 as well as the 10 aminoterminal residues of ATR16 and the 10 carboxyterminal residues of ATR17 are located in regions of ill-defined electron density [15]. Guided by sequence alignment, remaining ATR13, 14, 15, the 20 most carboxyterminal residues of ATR16 and the 11 most aminoterminal residues of ATR17 can be projected onto the three-dimensional structure of HSV-1 gD as illustrated in Figure 7.

ATR17 makes up the C-terminus of the ordered polypeptide chain (blue in Figure 7) and is packed crosswise on top of ATR13 (N-terminus of ordered chain, yellow in Figure 7). Both together form the crest of a protrusion from the gD molecules while ATR13, 14 and 15 contribute solvent-exposed areas on both of its flanks - with concomitant hiding of substantial parts of ATR sequence in the interior. A possible explanation for this is offered by assuming that, in the natural situation, alternative structures are available to the domain termini in which areas seen as buried in the crystal are (transiently ?) exposed and made accessible for interaction with B cell receptors. This kind of structural flexibility of the chain segments under consideration has been invoked earlier in connection with binding of gD to infection-relevant host receptors [15], [16]. In particular, contacts of gD to bound nectin-1 [16] involve extensive surface area of ATR15 buried in the closed conformation. Of note, in Figure 7, N and C do not represent true physical chain termini [15].

## Discussion

Prior to our study, only one epitope had been mapped to a defined site within any antigen of herpes B [8]. In contrast, scanning a few dozens of monkey sera with a tiled peptide microarray representing glycoproteins gB and gD was sufficient to identify and precisely locate 18 ATRs within these two polypeptides. Several ATRs of gD cluster in a locally confined region of the protein surface with obviously high antigenicity. In the chip assay, macaque sera reacting uniformly positive in HSV-ELISA individually responded to strikingly different, fingerprint-like subsets of the 18 ATRs.

### Immunological Impact of ATR Distribution

Although the majority of the identified ATRs are located on the outside of the glycoproteins, some ATRs are likely located on the inner side of the viral envelope amounting to 33% of all individual ATR-directed responses. This effect is particularly pronounced for ATR18, which alone accounts for ∼79% of all individual responses mapping to gD. This is in agreement with an earlier study that had identified the nine most carboxyterminal residues of gD as an immunodominant B cell epitope in rhesus macaques – hitherto the only one mapped to a specific position within an antigen of herpes B [8]. While antibodies directed against targets hidden inside a closed membrane are a rather common phenomenon, it is not clear how they arise.

For a protein like gD, with a single membrane-spanning domain and a short cytosolic tail, reversible switching of membrane topology as reported for HBV from TM1 to TM3 [17], thereby exposing parts of the endodomain is very unlikely. Still, ATR18 is highly antigenic but, significantly, it does not elicit neutralizing antibodies [8]. By comparison, the existence of neutralizing antibodies directed against the so-called Kennedy epitope of HIV-1 has been used as an argument supporting a topology switch of the (much larger) gp41 cytosolic tail [18]–[20]. The same issue has additionally been discussed in connection with the cytosolic tail of SIV gp41 and put in relation with other known cases [21].

The ectodomain of gB contains a conspicuously large gap devoid of ATRs. This is true for both amino acid sequence (compare e.g. Figure 2A, gap between ATR4 and ATR5) and the three-dimensional structure (Figure 6). Protein glycosylation is a plausible candidate for causing these gaps. Almost all known glycosylation sites of HSV-1 gB and gD are located outside the ATRs found during this present study [22], [23]. This may be due to two different, mutually non-exclusive reasons: *(i)* antibodies directed against glycosylated sites may be present in the serum but not able to bind to the corresponding unglycosylated peptides on the chip; *(ii)* glycans may shield surrounding areas of the protein from docking of B cell receptors [24].

Comparison of the here described ATRs in herpes B with published linear epitopes of HSV-1 and -2 [25], [26] reveals that some antigenic regions are very similar between these closely related viruses. As outlined in Table S1, linear epitopes that were described for HSV partially or completely overlap with ATRs 2, 3, 5, 12, 16 and 17. However, at least some of these lack high sequence similarity. For example, ATR2 completely overlaps with the known HSV gB epitope H1817 [26], but only 6 out of 13 amino acid residues are identical (Table S1). This might indicate that the structural position of this linear epitope is responsible for its immunogenicity rather than the primary amino acid sequence. Another factor contributing to different epitope patterns may be the divergent immune response of humans *versus* non-human primates.

### Structure of Peptide #197

As mentioned under Results, spot #197 represents an antibody target of 15 amino acid residues formal length located at the C-terminal end of ATR7. This has to be attributed to a single epitope since a single responding spot implies that removal of three amino acid residues from either end of the 15mer leads to complete loss of binding – and this is incompatible with the notion of a target region composed of two or more distinct epitopes. On the other hand, it seems difficult to imagine how that much polypeptide chain could be accommodated in the antigen binding pocket of a typical antibody. The location of ATR7 at the surface of gB (Figure 6) lends additional credibility to its being a true B cell epitope, but a convincing structural explanation is lacking. Intriguingly, the sequence stretch corresponding to peptide #197 (V589 - Q603) coincides with a β-hairpin resting on or protruding from the gB surface (Figure 6). The interior of the ATR7 sequence (i.e. the loop of the hairpin) could be irrelevant for antibody binding but the critical contacting residues be distributed among the two strands of the β -hairpin – well separated along the chain but close neighbors in space. Any shortening of peptide #197, at either end, would remove such critical residues and lead to loss of antibody binding. Alternatively, the critical residues could reside within a continuous stretch of loop sequence but binding of the antibody depends upon a particular chain conformation requiring the structural context of the β-hairpin. Formation of the latter would, in turn, be sensitive against any shortening of either of the two β-strands. As a corollary of the above, ATR7 emerges as a highly antigenic region in a special sense: It seems to elicit, with high selectivity (nine out of ten cases), antibodies that are conformation-dependent in one of two possible ways sketched above.

### Diagnostic Implications

Mapping anti-herpes B antibody targets may be a valuable tool to assist or confirm current HSV-ELISA based diagnostics of herpes B. Taking HSV-ELISA results as a reference, the peptide microarray chip assay produced 27% false negatives for gB and 15% for gD. The observed number of double failures was one – in accord with prediction made on the premise of gB and gD false negatives occurring independently. On this basis, a crude estimate can be made of the extent by which the rate of false negative results could be brought down further by incorporating additional antigens, e.g. gC and gD [4]. It can be calculated that with 12 relevant ATRs per antigen, a test based on less than 50 different peptides could bring the frequency of false negatives down to the range of one in a thousand. Since this number of peptides is manageable, a peptide ELISA- or bead-based diagnostic test could be developed as an alternative to possibly cost-limiting peptide microarrays.

### Limitations of the Method

Microarrays of tiled peptides delineating total amino acid sequences of relevant antigens are excellent tools to determine, in a single simple experiment, the entire spectrum of linear epitopes of that antigen recognized by antibodies populating a specific individual serum. As already mentioned above, glycosylation-specific antibodies will be missed as long as the microarrays do not include relevant peptide-glycan conjugates. The same is true for other post-translational protein modifications such as phosphorylation, acetylation and methylation. Furthermore, discontinuous epitopes will be missed. Solutions suggested in the literature [27], [28] include chips displaying binary combinatorial libraries of antigen fragments and of peptides conformationally constrained by cyclization. A more general solution to the above problems might be offered by replacing antigen-specific scanning chips by arrays of large numbers of peptides of random sequence.

### Outlook

The data documented and discussed above reinforce the unique usefulness of synthetic peptide microarray chips in characterizing antibody spectra at the level of individual epitopes or ATRs. Clearly, the potential of the method reaches beyond mere factual assessment of viral infections into direct and detailed visualization of individual humoral immune responses mounted against a pathogen or vaccine with potentially profound consequences for therapy, prognosis and monitoring vaccination or therapeutic success. Moreover, work in progress in our laboratories endorses high resolution chip serology as a tool for gleaning insight on issues of fundamental biological and medical importance such as viral multiplication, latency and escape.

## Supporting Information

Table S1 **Comparison of antibody target regions (ATRs) in glycoprotein B and D of herpes B virus with known epitopes of herpes simplex virus (HSV).**
(DOCX)

## References

[1] Sabin AB, Wright AM (1934) Acute ascending myelitis following a monkey bite, with the isolation of a virus capable of reproducing the disease. J Exp Med 59: 115–136. 19870235 10.1084/jem.59.2.115 PMC2132353

[2] Perelygina L, Zhu L, Zurkuhlen H, Mills R, Borodovsky M, et al (2003) Complete sequence and comparative analysis of the genome of herpes B virus (Cercopithecine herpesvirus 1) from a rhesus monkey. J Virol 77: 6167–6177. 12743273 10.1128/JVI.77.11.6167-6177.2003 PMC155011

[3] Jainkittivong A, Langlais RP (1998) Herpes B virus infection. Oral Surg Oral Med Oral Pathol Oral Radiol Endod 85: 399–403. 9574948 10.1016/s1079-2104(98)90064-6

[4] Elmore D, Eberle R (2008) Monkey B virus (Cercopithecine herpesvirus 1). Comp Med 58: 11–21. 19793452 PMC2703160

[5] Perelygina L, Patrusheva I, Hombaiah S, Zurkuhlen H, Wildes MJ, et al (2005) Production of herpes B virus recombinant glycoproteins and evaluation of their diagnostic potential. J Clin Microbiol 43: 620–628. 15695655 10.1128/JCM.43.2.620-628.2005 PMC548098

[6] Morton WR, Agy MB, Capuano SV, Grant RF (2008) Specific pathogen-free macaques: definition, history, and current production. ILAR J 49: 137–144. 18323576 10.1093/ilar.49.2.137

[7] Coulibaly C, Hack R, Seidl J, Chudy M, Itter G, et al (2004) A natural asymptomatic herpes B virus infection in a colony of laboratory brown capuchin monkeys (Cebus apella). Lab Anim 38: 432–438. 15479559 10.1258/0023677041958891

[8] Perelygina L, Zurkuhlen H, Patrusheva I, Hilliard JK (2002) Identification of a herpes B virus-specific glycoprotein d immunodominant epitope recognized by natural and foreign hosts. J Infect Dis 186: 453–461. 12195371 10.1086/341834

[9] Frank R (1992) Spot-synthesis - an easy technique for the positionally addressable, parallel chemical synthesis on a membrane support. Tetrahedron 48: 9217–9232.

[10] Dikmans A, Beutling U, Schmeisser E, Thiele S, Frank R (2006) SC2: A novel process for manufacturing multipurpose high-density chemical microarrays. Qsar & Combinatorial Science 25: 1069–1080.

[11] Schneider CA, Rasband WS, Eliceiri KW (2012) NIH Image to ImageJ: 25 years of image analysis. Nat Methods 9: 671–675. 22930834 10.1038/nmeth.2089 PMC5554542

[12] Perelygina L, Patrusheva I, Zurkuhlen H, Hilliard JK (2002) Characterization of B virus glycoprotein antibodies induced by DNA immunization. Arch Virol 147: 2057–2073. 12417944 10.1007/s00705-002-0889-0

[13] Gilbert R, Ghosh K, Rasile L, Ghosh HP (1994) Membrane anchoring domain of herpes simplex virus glycoprotein gB is sufficient for nuclear envelope localization. J Virol 68: 2272–2285. 8139012 10.1128/jvi.68.4.2272-2285.1994 PMC236703

[14] Heldwein EE, Lou H, Bender FC, Cohen GH, Eisenberg RJ, et al (2006) Crystal structure of glycoprotein B from herpes simplex virus 1. Science 313: 217–220. 16840698 10.1126/science.1126548

[15] Krummenacher C, Supekar VM, Whitbeck JC, Lazear E, Connolly SA, et al (2005) Structure of unliganded HSV gD reveals a mechanism for receptor-mediated activation of virus entry. EMBO J 24: 4144–4153. 16292345 10.1038/sj.emboj.7600875 PMC1356314

[16] Di Giovine P, Settembre EC, Bhargava AK, Luftig MA, Lou H, et al (2011) Structure of herpes simplex virus glycoprotein D bound to the human receptor nectin-1. PLoS Pathog 7: e1002277. 21980294 10.1371/journal.ppat.1002277 PMC3182920

[17] Bruss V (2007) Hepatitis B virus morphogenesis. World J Gastroenterol 13: 65–73. 17206755 10.3748/wjg.v13.i1.65 PMC4065877

[18] Hollier MJ, Dimmock NJ (2005) The C-terminal tail of the gp41 transmembrane envelope glycoprotein of HIV-1 clades A, B, C, and D may exist in two conformations: an analysis of sequence, structure, and function. Virology 337: 284–296. 15913700 10.1016/j.virol.2005.04.015 PMC7111842

[19] Steckbeck JD, Kuhlmann AS, Montelaro RC (2013) C-terminal tail of human immunodeficiency virus gp41: functionally rich and structurally enigmatic. J Gen Virol 94: 1–19. 23079381 10.1099/vir.0.046508-0 PMC3542723

[20] Steckbeck JD, Sun C, Sturgeon TJ, Montelaro RC (2010) Topology of the C-terminal tail of HIV-1 gp41: differential exposure of the Kennedy epitope on cell and viral membranes. PLoS One 5: e15261. 21151874 10.1371/journal.pone.0015261 PMC2998427

[21] Postler TS, Martinez-Navio JM, Yuste E, Desrosiers RC (2012) Evidence against extracellular exposure of a highly immunogenic region in the C-terminal domain of the simian immunodeficiency virus gp41 transmembrane protein. J Virol 86: 1145–1157. 22072749 10.1128/JVI.06463-11 PMC3255797

[22] Wang J, Fan Q, Satoh T, Arii J, Lanier LL, et al (2009) Binding of herpes simplex virus glycoprotein B (gB) to paired immunoglobulin-like type 2 receptor alpha depends on specific sialylated O-linked glycans on gB. J Virol 83: 13042–13045. 19812165 10.1128/JVI.00792-09 PMC2786847

[23] Sodora DL, Cohen GH, Eisenberg RJ (1989) Influence of asparagine-linked oligosaccharides on antigenicity, processing, and cell surface expression of herpes simplex virus type 1 glycoprotein D. J Virol. 63: 5184–5193. 10.1128/jvi.63.12.5184-5193.1989 PMC251182 2555549

[24] Vigerust DJ, Shepherd VL (2007) Virus glycosylation: role in virulence and immune interactions. Trends Microbiol 15: 211–218. 17398101 10.1016/j.tim.2007.03.003 PMC7127133

[25] Lazear E, Whitbeck JC, Ponce-de-Leon M, Cairns TM, Willis SH, et al (2012) Antibody-induced conformational changes in herpes simplex virus glycoprotein gD reveal new targets for virus neutralization. J Virol 86: 1563–1576. 22130533 10.1128/JVI.06480-11 PMC3264331

[26] Bender FC, Samanta M, Heldwein EE, de Leon MP, Bilman E, et al (2007) Antigenic and mutational analyses of herpes simplex virus glycoprotein B reveal four functional regions. J Virol 81: 3827–3841. 17267495 10.1128/JVI.02710-06 PMC1866100

[27] Timmerman P, Beld J, Puijk WC, Meloen RH (2005) Rapid and quantitative cyclization of multiple peptide loops onto synthetic scaffolds for structural mimicry of protein surfaces. Chembiochem 6: 821–824. 15812852 10.1002/cbic.200400374

[28] Timmerman P, Van Dijk E, Puijk W, Schaaper W, Slootstra J, et al (2004) Mapping of a discontinuous and highly conformational binding site on follicle stimulating hormone subunit-beta (FSH-beta) using domain Scan and Matrix Scan technology. Mol Divers 8: 61–77. 15209158 10.1023/b:modi.0000025650.94399.bb

