# Supplementary material for: Multiple Antibody Targets on Herpes B Glycoproteins B and D Identified by Screening Sera of Infected Rhesus Macaques with Peptide Microarrays
Source: PLoS One. 2014 Jan 31;9(1):e86857. doi: 10.1371/journal.pone.0086857 (PMC3908960; doi:10.1371/journal.pone.0086857)
Supplement: Table S1 — Comparison of antibody target regions (ATRs) in glycoprotein B and D of herpes B virus with known epitopes of herpes simplex virus (HSV). (DOCX) [file pone.0086857.s001.docx]

| **Supplementary Table S1:** Comparison of antibody target regions (ATRs) in glycoprotein B and D of herpes B virus with known epitopes of herpes simplex virus (HSV) | | | | | | |
| --- | --- | --- | --- | --- | --- | --- |
|  |  |  |  |  |  |  |
| **ATR** | **Spot no.** | **HSV MAb** | **aa position** | **aa sequence *** | **% identity**  **between herpes B**  **and HSV-1** | **aa length** |
|  | | | | | | |
| **glycoprotein B** | | | | | | |
| 1 | 2 - 3 |  | 4 - 21 | RAGPLPLPSPLVPLLALA | 16.7 | 18 |
|  |  | - | - |  |  |  |
| 2 | 10 - 14 |  | 28 - 54 | **P**LG**P**-----**AAAT**PVVSPRASPAPPVPAATPT | 48.2 | 27 |
|  |  | H1817 ¹ | 31 - 43 | **P**SS**P**GTPGV**AAAT** | 46.2 ² | 13 |
| 3 | 16 - 21 |  | 46 - 75 | PPVPAATPTF**P**DDDNDGEAGAAPGAPGTNA | 36.7 | 30 |
|  |  | B6 ¹ | 68 - 76 | K**P**KKNRKPK | 11.1 ² | 9 |
| 4 | 26 - 28 |  | 76 - 96 | SVEAGHATLRENLRDIKALDG | 71.4 | 21 |
|  |  | - | - |  |  |  |
|  |  | - | - |  |  |  |
|  |  | H1838 ¹ | 391 - 410 | ISTTFTTNLTEYPLSRVDLG | 85.0 | 20 |
| 5 | 151 - 154 |  | 451 - 474 | **YVRE**L**LREQ**E**R**RPGDAAATPKPSA | 45.8 | 24 |
|  |  | H1781 ¹ | 454 - 473 | LLSNTLAEL**YVRE**H**LREQ**S**R** | 80.0 ² | 20 |
| 6 | 157 - 159 |  | 469 - 489 | TPKPSADPPDVERIKTTSSVE | 52.4 | 21 |
|  |  | - | - |  |  |  |
| 7 | 196 - 197 |  | 586 - 603 | RPLVSFRYEEGGPLVEGQ | 88.9 | 18 |
|  |  | - | - |  |  |  |
|  |  | - | - |  |  |  |
|  |  | Group V ¹ | 697 - 716 | SGLLDYTEVQRRNQLHDLRF | 95.0 | 20 |
|  |  | - | - |  |  |  |
|  |  | Group V ¹ | 706 - 725 | QLHDLRFADIDTVI | 90.0 | 20 |
| 8 | 239 - 242 |  | 715 - 738 | HAALFAGLYSFFEGLGDVGRAVGK | 75.0 | 24 |
|  |  | - | - |  |  |  |
| 9 | 273 - 275 |  | 817 - 837 | DGASGGGEEDFDEAKLAQARE | 66.7 | 21 |
|  |  | - | - |  |  |  |
| 10 | 284 - 288 |  | 850 - 876 | RTEHKARKKGTSALLSAKVTNMVMRKR | 92.6 | 27 |
|  |  | - | - |  |  |  |
| 11 | 292 - 294 |  | 874 - 892 | RKRAKPRYSPLGDTDEEEL | 47.4 | 19 |
|  |  | - | - |  |  |  |
|  | | | | | | |
| **glycoprotein D** | | | | | | |
| 12 | 392 - 396 |  | 22 - 48 | GGGEYVPVERSLTRVN**P**G**RFRG**AHLAP | 37.0 | 27 |
|  |  | Group VII | 35 - 45 | KMAD**P**N**RFRG**K | 45.5 ² | 11 |
| 13 | 401 - 402 |  | 49 - 66 | LEQKTDPPDVRRVYHVQP | 72.2 | 18 |
|  |  | - | - |  |  |  |
| 14 | 436 |  | 154 - 168 | VRNLPRWSFYDNFGA | 53.3 | 15 |
|  |  | - | - |  |  |  |
| 15 | 465 - 466 |  | 244 - 258 | GIGMLPRFIPENQRIVAV | 93.3 | 18 |
|  |  | - | - |  |  |  |
|  |  | - | - |  |  |  |
|  |  | Group XIV ³ | 260 - 270 | SLKIAGWHGPK | 18.2 | 11 |
| 16 | 479 - 484 |  | 283 - 312 | VETA**NAT**R**PELAPED**EDEQ**A**PGDEPAPAVA | 53.3 | 30 |
|  |  | Group Iia ³ | 287 - 297 | P**NAT**Q**PELAPE** | 81.8 ² | 11 |
|  |  | Group Iib ³ | 297 - 304 | **ED**PEDS**A**LL | 33.3 ² | 9 |
| 17 | 492 - 494 |  | 322 - 342 | **P**EAS**D**VTIQGPA**PA**PSGHTGA | 23.8 | 21 |
|  |  | Group XI ³ | 305 - 341 | EDPVGTVAPQIPPNWHI**P**SIQ**D**AATPYHP**PA**TPNNM | 21.1 ² | 37 |
| 18 | 507 - 512 |  | 367 - 394 | RARAAGKHVRLPELLDEGPGPARRGAPY | 21.4 | 28 |
|  |  | - | - |  |  |  |

* identical amino acid residues are labeled in bold; ¹ Bender *et al.,* 2007 [26]; ² identity of overlapping amino acid (aa) sequence; ³ Lazear *et al.,* 2012 [25].
